# A Holographic Augmented Reality Guidance System for Patient Alignment: A Feasibility Study

**DOI:** 10.7759/cureus.14695

**Published:** 2021-04-26

**Authors:** Rex Cardan, Elizabeth L Covington, Richard Popple

**Affiliations:** 1 Radiation Oncology, University of Alabama at Birmingham, Birmingham, USA

**Keywords:** radiation therapy, augmented reality, hololens

## Abstract

Purpose

To evaluate the accuracy of an augmented reality holographic guidance system for potential use in patient alignment in radiotherapy applications.

Methods

A cubic phantom was scanned on a CT simulator and a 3D mesh was extracted using the Eclipse Scripting API. An application was created for the Microsoft HoloLens to allow users to see the scanned mesh as a hologram overlaid in the treatment vault. Six therapists were equipped with the HoloLens glasses and instructed to move the real phantom to align with the perceived spatial hologram using only couch controls. The initial couch coordinates were recorded and then recorded at each step as the therapist moved the phantom to each new location. The application varied the position of the virtual phantom to 10 preprogrammed locations within a 40-cm cubic volume in a combination of vertical, longitudinal, and lateral axis shifts. The absolute position difference between the holographic world and real-world phantom was recorded at each step. Also, the relative position from one position to the next was recorded.

Results

Fifty shifts were collected across the six therapists. The mean difference between the physical position and instructed holographic position was 0.58 ± 0.31 cm for relative shifts and 0.51 ± 0.33 cm for absolute position. The maximum difference between the holographic position and the actual post shift position was 1.53 cm for relative and 1.58 cm for absolute.

Conclusion

Holographic augmented reality guidance using the Microsoft HoloLens provides adequate accuracy for initial treatment alignment but lacks the fine alignment accuracy of X-ray imaging systems.

## Introduction

In recent years, the growth of computer vision, depth cameras, and computer graphics has enabled a new range of devices for virtual and augmented reality (AR) applications. Rather than virtual reality (VR), which fully immerses the user into a synthetic environment, AR overlays computer-generated objects over a real-world view [1]. Both VR and AR have been utilized in medicine with applications such as image-guided biopsy [2], surgery [3,4], and training purposes [5-7].

While some have explored the use of AR applications in radiotherapy [8-9], none have taken advantage of the most modern devices. This work explores the use of a new head-mounted AR device, the HoloLens (Microsoft, Redmond, WA), to aid therapists in aligning patients for treatment. We overview the workflow for the development of a holographic alignment system and evaluate the initial accuracy of the retail device using a phantom.

The HoloLens utilizes see-through holographic lenses to allow users to see their physical environment with holographic overlays. The device contains an infrared depth camera similar to the Kinect [10] (Microsoft, Redmond, WA), a 2-megapixel color camera, accelerometer, gyroscope, and magnetometer to help determine orientation relative to the user’s surroundings. By maintaining a local coordinate system at a rate of approximately 90 Hz, the unit is able to project images on a semi-transparent glass surface providing an illusion that the projected images are registered to real-world objects in the user’s vision. The HoloLens operates as a standalone unit containing its own processor, RAM, battery, and Windows 10 operating system, making it ideal for portable operation.

This study was presented as a poster presentation at the 2017 AAPM Annual Meeting, July 30 to August 3, Denver, CO.

## Materials and methods

A cubic phantom was scanned in a similar manner of patients for treatment using a computed tomography (CT) simulator. The images were transferred to the treatment planning system. Using the Eclipse Scripting API (application programming interface) (Varian Medical Systems, Palo Alto, CA), a 3D triangle mesh was extracted of the phantom object. This mesh asset was then used as a holographic guide inside the virtual world of the HoloLens.

**Figure 1:**
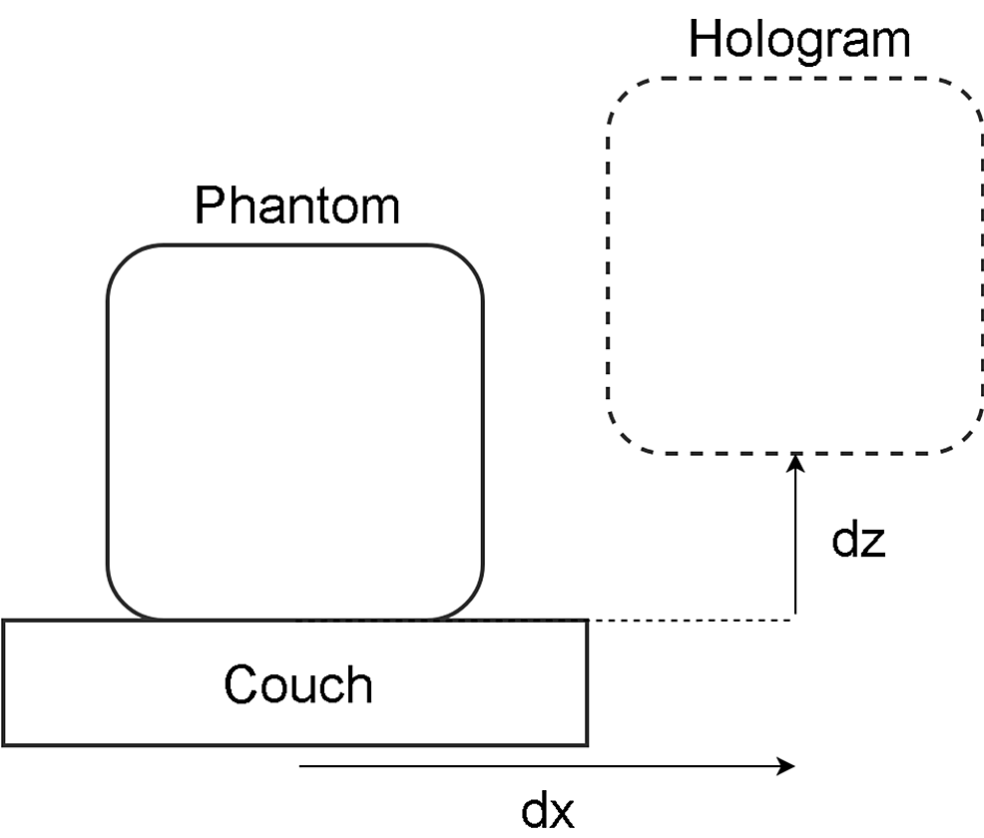
A hologram of the phantom is visible through the HoloLens device and is used to guide a physical phantom to the desired location by moving the couch until they are aligned.

A simple application was created in Unity, the development environment for HoloLens, which allowed the initial placement of the virtually projected phantom as well as instructions for the movement of the phantom from the initial position. This virtual phantom, when visualized through the HoloLens, could then be used to align a physical object and simulate a patient positioning workflow. The application could display an AR outline of a physical object in the users' view, and the user could move a real object until it aligned with the virtual one (Figure 1).

**Figure 2:**
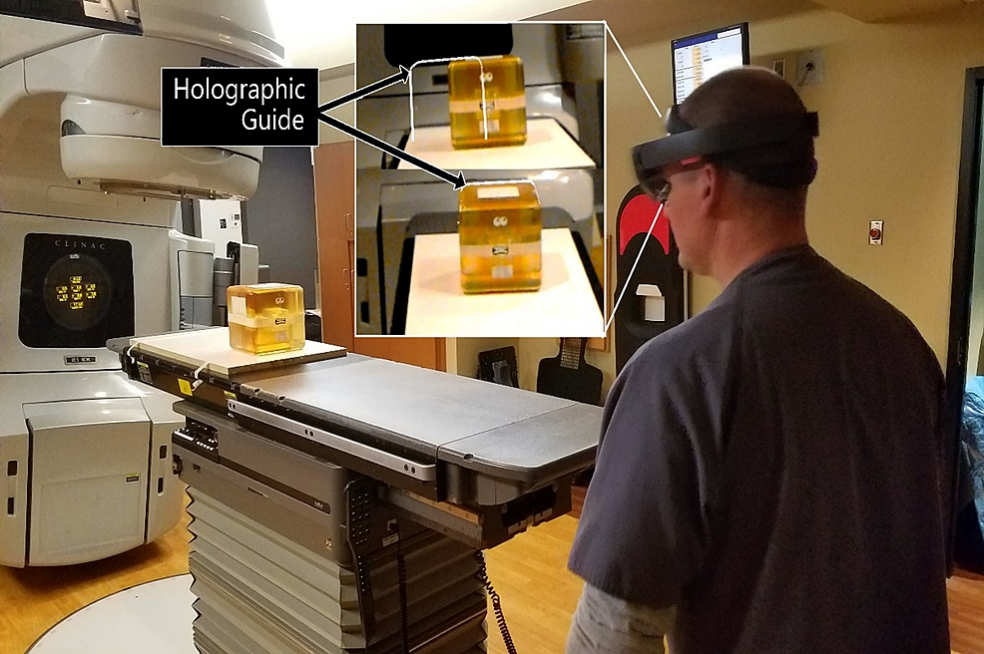
A therapist adjusts the couch positions to align a physical phantom to a holographic outline, visible to the user as a 3D glowing cube, using the Microsoft HoloLens. The top image shows that the current alignment is off, and the lower image shows a perfectly aligned phantom.

Using the application, a physicist placed the virtual phantom on the couch top. Once the virtual object was placed, a therapist was equipped with the HoloLens device. The virtual phantom, visible as a 3D glowing cube, was then used by the therapist to align the actual physical phantom by adjusting the couch positions until the holographic image was overlaid with the actual phantom position, as shown in Figure 2. Once the therapist aligned to the best of their ability, this position was considered the baseline position.

The application then varied the position of the virtual phantom to 10 preprogrammed locations within a 40-cm cubic volume in a combination of vertical, longitudinal, and lateral axis shifts. Upon each move, the therapist was instructed to move the physical phantom to the holographic position using only the couch controls. At each iteration, the couch parameters were recorded. The couch controls allowed for precise quantification of the vector offsets from therapist movements and also allowed the move to be performed at a distance.

To assess the accuracy of motion following holographic instruction, two metrics were considered: relative and absolute offsets. Relative offsets were the offsets from the previous position to the current position and ignored any aggregate errors from prior shifts. Absolute offset was the total vector offset from the initial baseline position to the current position, including all aggregate errors from prior shifts.

## Results

**Figure 3:**
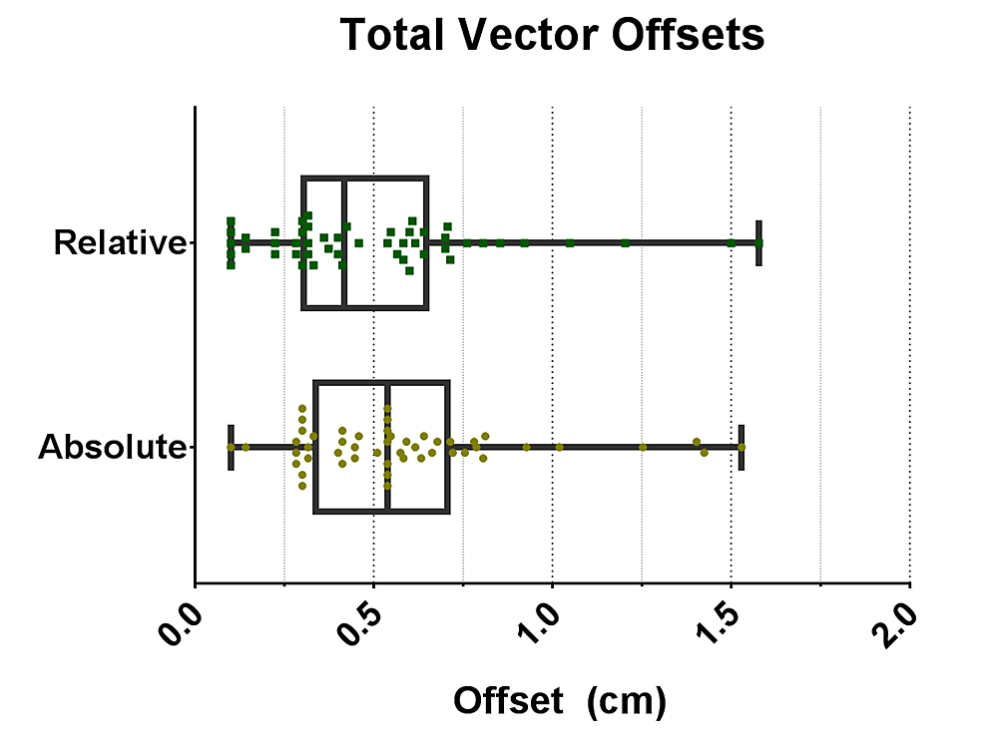
Distribution of the absolute vector offset between the holographic instructed position and the actual post shift position made by each therapist. The box plot shows the minimum, maximum, 25%, 50%, and 75%.

**Figure 4:**
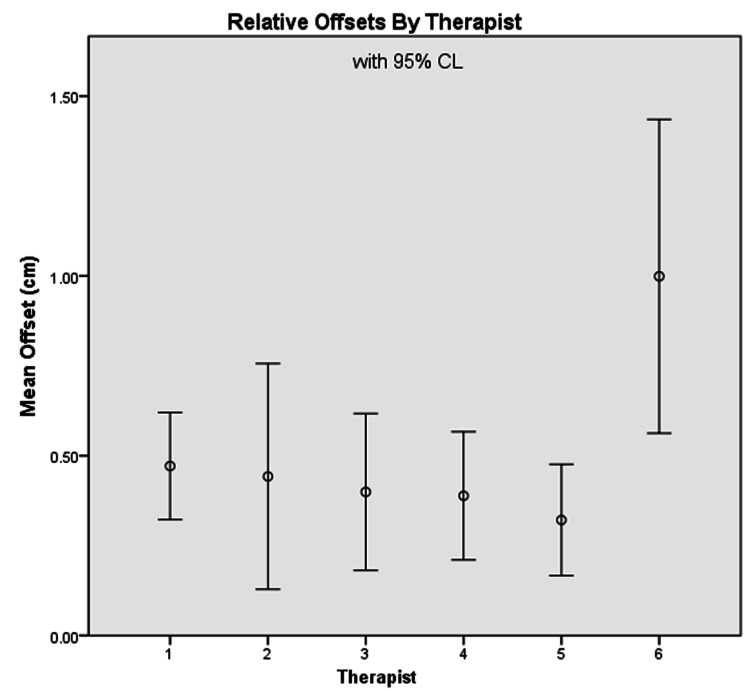
Distribution of relative offsets between the various therapists. The offset represents the difference between the instructed holographic position and the actual position moved.

Six therapists performed 50 shifts that were within the range of the couch motions. The mean difference between the physical position and instructed holographic position was 0.58 ± 0.31 cm for relative shifts and 0.51 ± 0.33 cm for absolute position. The maximum difference between the holographic position and the actual post shift position was 1.53 cm for relative and 1.58 cm for absolute. Figure 3 shows the total vector offsets for both the absolute and relative motions, while Figure 4 shows the mean offsets of all positions moved by each individual therapist.

## Discussion

The advance of computer graphics in VR and AR is moving at a rapid pace. The hardware capabilities created by the HoloLens allow for virtual objects to be superimposed over real-world objects from a user's viewpoint. The device uses real-time registration of the environment without external tracking sensors. Instead, the coordinate system is generated by triangulation of local surfaces while maintaining a map of each surface from frame to frame in a technique called "inside-out tracking" [11].

This tracking mechanism, while easy to set up and probably appropriate for more general use cases, appears to be inadequate for high accuracy setup required in radiation oncology. In this study, the magnitude of error was approximately one order of magnitude larger than commercial surface imaging systems [12]. One problem that we noticed is the coordinate system changed slightly while components in the room (i.e., gantry, couch) were in motion. Other tracking systems with fixed reference points [13] would be more appropriate for AR applications. A fixed reference could aid in stability of the coordinate system and be made to coincide with the inherit treatment coordinates of the treatment room. We believe that this is the main limitation of using the device in a clinical scenario. The results of this work would likely be dramatically improved if a fixed reference system was made available.

AR has been previously investigated as a means to align patients for radiotherapy treatments [9]. Talbot et al. created an AR system using cameras placed within the vault to capture a live feed of the patient. A 3D representation of a phantom’s contour was superimposed over the video feed and displayed on a laptop computer. Unlike the HoloLens, this system requires the use of black and white planar makers placed in the room to allow for the registration of the room and computer-generated objects. In their initial camera configuration, the 3D error vector was 5.9 mm, which is comparable to the average offsets found in this study. They were able to reduce their error to 3.0 mm by changing the locations of their cameras. Our larger range in offsets can be attributed to two causes. First, our study contains over 50 measurements, while the aforementioned study was a proof-of-principle study and only performed one set-up per each camera configuration. Secondly, their study was performed with one user who was an experienced with the equipment. Our study used therapists who had no knowledge of the procedure and had never previously used the HoloLens device.

During alignment, a live feed of the HoloLens was streamed on a laptop that was viewed by a physicist. The physicist was able to observe the accuracy of placement between users but did not provide feedback or influence the results. As shown in Figure 4, spread in the alignment accuracy is attributed to variations in accuracy of each user's alignment. This is attributed to the therapist's comfort level with using the technology and the fit of the HoloLens device. For the best performance, HoloLens has a process that optimizes the display for a specific user. This process was not repeated for each user; therefore, this could be the cause of variations between users. For clinical use, therapists would be trained before using the system and have uniquely registered devices that ostensibly would reduce the error.

## Conclusions

Holographic AR guidance using the Microsoft HoloLens provides adequate accuracy for initial treatment alignment but lacks the fine alignment accuracy of X-ray imaging systems. The introduction of a fixed reference system would likely improve the performance to levels more appropriate for robust clinical setups. Future work includes integrating in-room markers to establish absolute coordinates, using anthropomorphic phantoms, and sampling from a larger cohort of users.
